# Identification and Evaluation of Microplastics from Tea Filter Bags Based on Raman Imaging

**DOI:** 10.3390/foods11182871

**Published:** 2022-09-16

**Authors:** Tingna Mei, Jiahua Wang, Xiaofeng Xiao, Jingwen Lv, Qiaocong Li, Huang Dai, Xiaodan Liu, Fuwei Pi

**Affiliations:** 1 College of Food Science and Engineering, Wuhan Polytechnic University, Wuhan 430023, China; 2 Hubei Key Laboratory for Processing and Transformation of Agricultural Products, Wuhan Polytechnic University, Wuhan 430023, China; 3 School of Food Science and Technology, Jiangnan University, Wuxi 214122, China

**Keywords:** plastic-based filter bag, microplastics, Raman imaging, risk assessment, origin exploration

## Abstract

Microplastic (MP) contamination is a public issue for the environment and for human health. Plastic-based food filter bags, including polyethylene terephthalate, polypropylene, nylon 6 (NY6), and polyethylene, are widely used for soft drink sub-packaging, increasing the risk of MPs in foods and the environment. Three types of commercially available filter bags, including non-woven and woven bags, were collected, and MPs released after soaking were mapped using Raman imaging combined with chemometrics. Compared with peak area imaging at a single characteristic peak, Raman imaging combined with direct classical least squares calculation was more efficient and reliable for identifying MP features. Up to 94% of the bags released MPs after soaking, and there was no significant correlation with soaking conditions. Most MPs were tiny fragments and particles, and a few were fibrous MPs 620–840 μm in size. Woven NY6 filter bags had the lowest risk of releasing MPs. Source exploration revealed that most MPs originated from fragments and particles adsorbed on the surface of bags and strings. The results of this study are applicable to filter bag risk assessment and provide scientific guidance for regulating MPs in food.

## 1. Introduction

Microplastic (MP) (size < 5 mm) contamination is a public issue for the environment and human health [1]. Due to the tiny size of MPs, they easily pass through the gastrointestinal tract after being ingested by humans. In particular, when the particle size is less than 10 μm, MPs can cross cell membranes and reach the circulatory system [2]. Several studies have reported the observation of MPs in human stool [3], colectomy specimens [4], urine [5], blood [6], placenta, and meconium [7]. Of even greater concern is the fact that these tiny MPs can be transmitted to young infants through the placenta and breast milk [8]. Toxicological studies have confirmed that micro-nanoplastics accumulated in the intestine, liver, and kidney cause oxidative stress, energy deficiency, lipid disturbance, and neurotoxicity in mammals (mice) [9]. In addition, in vitro cytotoxicity assays have also shown that nanoplastics induce inflammation and genotoxicity in different human hematopoietic cell lines [10]. In the past, researchers have paid more attention to the impact of MPs in the environment on food safety and human health [11], but less exploration has been done on the risk assessment of MPs released from food packaging materials, especially single-use filter bags.

One of the sources of MP accumulation in the human body is the ingestion of food contaminated with MPs from the environment [12,13,14,15,16,17,18,19,20,21]. Another source is the release of large amounts of MPs from plastic packaging materials that come into direct contact with food, which has been confirmed by researchers. For example, MPs are shed from disposable plastic-based packing containers during washing and soaking [22,23,24]. Weisser et al. found that plastic bottles and caps were the main sources of MPs in single-use bottled mineral water [25]. Multiple autoclaving procedures used in baby bottles led to the shedding of MPs from the bottle surface and into infant formula [26]. The study also confirmed that when polyethylene corks were used for packaging white wines, MPs were transferred from corks to white wines during storage and transportation [27]. Thus, monitoring the release of MPs in food packaging and containers is necessary to provide scientific guidance for people to avoid or control the intake of MPs.

Currently, several techniques have been developed for qualitative identification and quantitative assessment of MPs [28]. Among them, the simplest approach is microscopic techniques, such as scanning electron microscopy coupled with energy dispersive X-ray spectroscopy. This approach can directly observe the MPs, and meanwhile, it could identify the elemental composition. Although this method is simple, the MPs cannot be chemically identified [29]. Recognition of MPs’ features, e.g., their thermal responses and specific chemical groups, is another approach to evaluate the contamination of MPs. Thermal analysis has a high sensitivity to MPs (<50 nm) and can provide accurate information for polymer identification; however, information on the size and shape of the particles cannot be obtained [18]. Different from the physical observations and evaluations, vibrational spectroscopy, i.e., infrared (IR) and Raman spectroscopy, provides information on the chemical composition in the sample. Fourier transform IR (FTIR), including attenuated total reflection-FTIR (ATR-FTIR) and micro-FTIR, can detect MPs above 10 μm, while Raman can detect MPs above 1 μm. Moreover, focal plane array-FTIR imaging can effectively detect the MPs larger than 20 μm [30], while Raman imaging can effectively identify MPs larger than 1 μm [31]. In addition to the inherent chemical information, Raman and IR spectroscopy combined with photothermal techniques provided high-resolution imaging for submicron-scale organic particle determination. For example, IR photothermal heterodyne imaging, optical photothermal IR and Raman spectroscopy can detect 400 nm particles [32,33].

Filter bags of different plastic substrates are widely used for the sub-packaging of soft drinks consumed after soaking, such as tea and coffee [34]. During soaking, MPs inevitably transfer from the filter bags to the water solution. Therefore, establishing a reliable testing method to identify different MPs simultaneously is of great significance for assuring the safe consumption and quality control of food packaged in filter bags. The present study aims to construct an efficient method based on Raman imaging, combined with chemometrics, for the simultaneous identification of various MPs released from filter bags during soaking. The specific objectives were to characterize different MPs by extracting features from Raman spectra; to combine Raman image data with chemometric methods to simultaneously identify various MPs; and to explain the origin of MPs from filter bags.

## 2. Materials and Methods

### 2.1. Samples and Pretreatment

**Figure 1:**
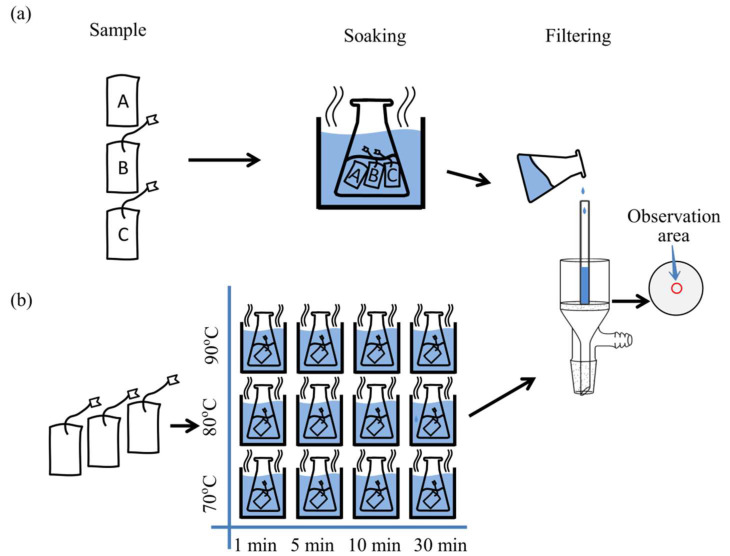
Sample preparation process for (**a**) optimization of peak area imaging for various MPs and (**b**) simultaneous identification of various MPs released from filter bags. A, B, and C in (**a**) represent sample A, sample B, and sample C, respectively.

Three types of food filter bags that are commercially available and usable in food packaging were labeled as non-woven polyethylene terephthalate (PET) (sample A without string), non-woven polypropylene (PP) (sample B with string), and woven nylon 6 (NY6) (sample C with string) and purchased on an e-commerce platform. Ultrapure water was used throughout the experiment to avoid interference from MPs in water. The filter bag samples were divided into two groups for Raman imaging optimization and detection method validation. Three samples (one of each type) were placed in the same conical flask and soaked in 30 mL ultrapure water at 90 °C for 12 h (Figure 1a). Thirty-six samples were randomly selected from three types of filter bags (12 of each type) and soaked in 30 mL ultrapure water at 70, 80, and 90 °C, respectively, and soaked for 1, 5, 10, and 30 min at each temperature (Figure 1b).

### 2.2. MP Separation

After soaking, the filter bag was taken out immediately, and the soaking solution was filtered using a vacuum extraction and filtration device. To collect the MPs released from the filter bag, a 47 mm diameter glass microfiber filter (GMF) with pore size of 0.7 μm (Whatman, London, UK) was used to separate the soaking liquid. The soaking solution was filtered through a Brinell funnel connected to a vacuum pump. A glass tube (diameter was ca. 9 mm) was applied to concentrate the MPs (Figure 1). In order to collect the MPs adsorbed on the inner wall of the glass tube, the glass tube was rinsed three times with ultrapure water after filtration. Throughout the experiments, dust-free glass Petri dishes and powder-free nitrile gloves were used, and all samplers were rinsed thoroughly with ultrapure water and wrapped in aluminum foil before use.

### 2.3. Raman Spectra and Images Acquisitions

All operations to acquire Raman data were implemented using Wire 5.3 (Renishaw Co., London, UK). Raman spectra and images were collected by a laser confocal micro-Raman system (model inVia Qontor; Renishaw Co., London, UK) equipped with a Leica microscope system (including a 50× long working distance objective and a 10× eyepiece lens), a 785 nm laser (rated power was ca. 300 mW), a 1200 lines/mm diffraction grating, and a cooled charge-coupled detector. The Raman spectra in the range of 100 to 3200 cm^−1^ were recorded once, and the exposure time and the exposure power were set to 10 s and 10%, respectively.

Raman images of GMFs were acquired by Stream high resolution (HR) scanning mode centered at 1300 cm^−1^ (spectral range of 800 to 1800 cm^−1^), with an exposure time of 0.3 s and exposure power of 10%. For Raman imaging, the size of the imaging area and the scanning step can be set as required. In order to overcome the change in focal plane caused by the size of tiny MPs and glass microfibers, real-time tracking technology was implemented on GMFs to obtain clear Raman images [35]. The confocal plane of the laser beam could automatically focus on the uneven sample surface so that every pixel was focused on the sample surface. Thus, the strongest Raman signal could be captured. When the selected imaging area was too large, the time for pixel-by-pixel scanning was long. For example, when the scanning step and exposure time were set to 3 µm and 0.3 s, respectively, 13.5 h were required for Raman imaging in an area of 1200 µm × 1200 µm. In the present study, one or several regions were selected from the observation area as the region of interest (ROI) for Raman imaging.

### 2.4. Date and Image Processing

Raman spectra and image analyses were conducted in Wire 5.3 software (Renishaw Co., London, UK). A series of preprocessing procedures were used to obtain high-quality spectra and images with a high signal-to-noise ratio. The spike removal method was proposed to remove cosmic rays, which were random and most likely came from the spectrometer itself. A polynomial fitting method was applied to correct the baseline. Noise filtering was performed to reduce the level of random noise in the dataset while preserving important spectral changes of interest.

After cosmic ray removal, baseline correction, noise reduction preprocessing, and peak area imaging based on a single characteristic peak were used to map MPs. Peak area imaging was performed by extracting the integration of peak intensity at characteristic peaks with a baseline. Additionally, a multivariate-based analysis method, direct classical least squares (DCLS) [36,37], was proposed for the identification and mapping of MPs. The DCLS fitting was performed with the Wire 5.3 software. The scale factors for all reference spectra were output under best-fit conditions, and the Raman images were segmented by thresholding the scale factors to create a map of the distribution of MPs. As each Raman spectrum is a fingerprint of the chemical composition of the sample, the spatial distribution of different substances can be analyzed. By assigning a color to each class of MP and color-coding the image, the distribution of all MPs within the observed area can be indicated.

The individual MP images were merged using ImageJ 1.8 software (National Institutes of Health, Bethesda, Rockville, MD, USA) so that a single map could present a variety MPs simultaneously.

## 3. Results and Discussion

### 3.1. Raman Spectral Properties of Different Filter Bags

In total, 36 specimens for three kinds of untreated filter bags (12 samples were randomly selected for each kind of bag) were analyzed with Raman spectroscopy to identify the compositions of the filter bag. Two regions were randomly selected on both sides of the filter bag, and two regions were randomly selected on the string as test points to collect Raman spectra. The Raman spectra of the filter bag and string were baseline-corrected and then used to calculate the mean spectrum. The mean Raman spectra of filter bags and strings were compared with the polymeric materials database in Wire 5.3 (Renishaw Co., London, UK).

**Figure 2:**
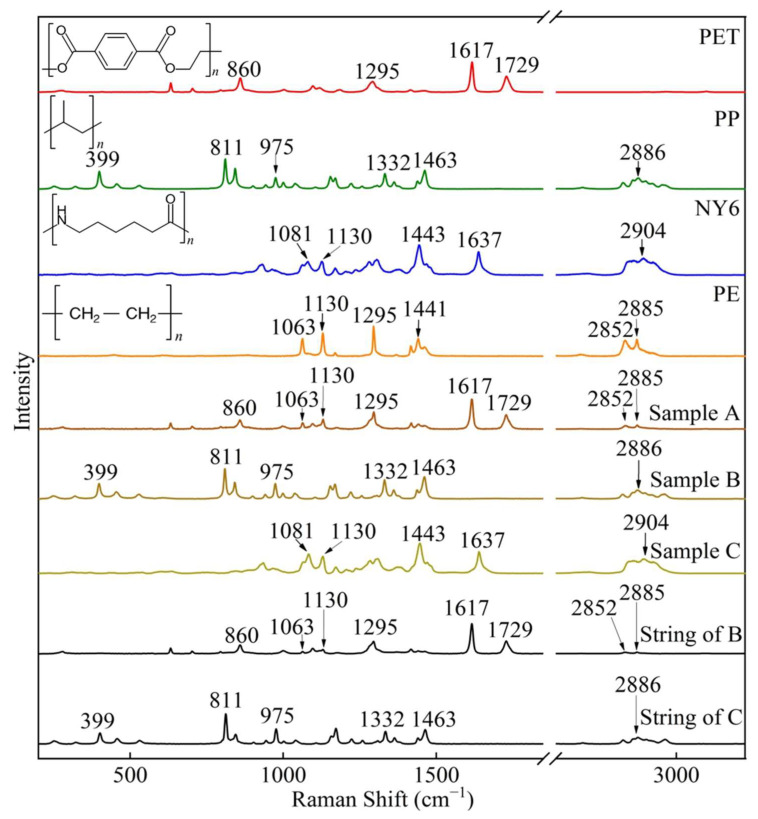
Raman spectra of the standards, filter bags, and strings. PET, PP, NY6, and PE were standard Raman spectra in database. Sample A, Sample B, Sample C, String of B, and String of C represent the mean spectra.

As plotted in Figure 2, peaks at 860, 1063, 1130, 1295, 1617, 1729, 2852, and 2885 cm^−1^ were observed in sample A. Compared with the standard database, sample A showed not only the main characteristic peaks of PET at 860, 1295, 1617, and 1729 cm^−1^ but also peaks at 1063, 1130, 2852, and 2885 cm^−1^, which highly correspond to the polyethylene (PE). After fitting the calculation, the matching ratios of sample A with PET and PE were 56.54% and 43.46%, respectively. These results are consistent with a previous study on plastic teabags primarily made of multiple materials [38]. The two intense peaks at 1617 and 1729 cm^−1^ were assigned to C–C vibration of the benzene ring and C=O stretching, respectively [39]. The peak at 860 cm^−1^ was related to ring C–C and C(O)–O stretching. The peak at 1295 cm^−1^ was associated with the stretching of C(O)–O bonds [40]. The peaks at 1063 and 1130 cm^−1^ were assigned to the anti-symmetric (ν_as__ym_(C–C)) and symmetric (ν_s__ym_(C–C)) stretching vibrations of the C–C bonds of PE, respectively. The peaks at 2852 and 2885 cm^−1^ belonged to the symmetric (ν_s__ym_(CH_2_)) and anti-symmetric (ν_as__ym_(CH_2_)) stretching vibrations of the CH_2_ groups of PE [41], respectively.

The Raman spectrum of sample B showed peaks at 399, 811, 975, 1332, 1463, and 2886 cm^−1^, and the matching rate with PP was 99.99% when compared with the standard database. As plotted in Figure 2, the peak at 399 cm^−1^ was assigned to CH_2_ vibration and CH bending. The intense peak at 811 cm^−1^ was assigned to CH_2_ rocking and stretching of C–C and C–CH_3_. The peak at 975 cm^−1^ was attributed to CH_3_ rocking and C–C stretching. The peak at 1332 cm^−1^ was attributed to CH bending and CH_2_ twisting, and the peak at 1463 cm^−1^ was ascribed to the anti-symmetric bending of CH_3_ and CH_2_ bending. Finally, the peak at 2886 cm^−1^ was assigned to symmetric stretching of the CH_3_ group in PP polymer [42,43].

Similarly, the Raman spectrum of sample C had peaks at 1081, 1130, 1443, 1637, and 2904 cm^−1^, which are 99% matched with the Raman spectrum of NY6. The peak at 1637 cm^−1^ was related to C=O stretching, which is characteristic of NY6 [39]. The peak at 1081 cm^−1^ was related to C–C stretching [44], and the peak at 1443 cm^−1^ was assigned to CNH bending [44]. The peak at 2904 cm^−1^ was assigned to the stretching of the CH_2_ group [39].

In addition, it can be seen from Figure 2 that the characteristic peaks of the string of sample B were completely consistent with the filter bag of sample A, which was made of PET and PE with a matching rate of 57.2% and 42.8%, respectively. In addition, the Raman spectrum of the string of sample C had a matching rate of 99.99% with polypropylene (PP) when compared with the standard database.

Based on the above observations, it can be reasonably inferred that the composition of the filter bags and strings were not completely consistent with the labels, and most of them contained two plastic components. The main characteristic peaks and assignments of filter bags, strings, and standards are listed in Table S1. Therefore, regardless of the plastic components of filter bags and strings, there is a risk of releasing MPs during soaking.

### 3.2. Optimization of Peak Area Imaging for Various MPs

A GMF coated with MPs was obtained following the soaking procedure (Figure 1a). An ROI was selected randomly from the observation area of the GMF for optimization of the scanning step during Raman imaging. Raman imaging was performed under different scanning steps of 1, 3, and 5 μm (Figure S1), and the appropriate scanning step was determined by comparative analysis. A scanning step of 3 µm was chosen for subsequent Raman imaging because it was less time-consuming than 1 μm resolution and the image contrast was better than at 5 μm resolution.

**Figure 3:**
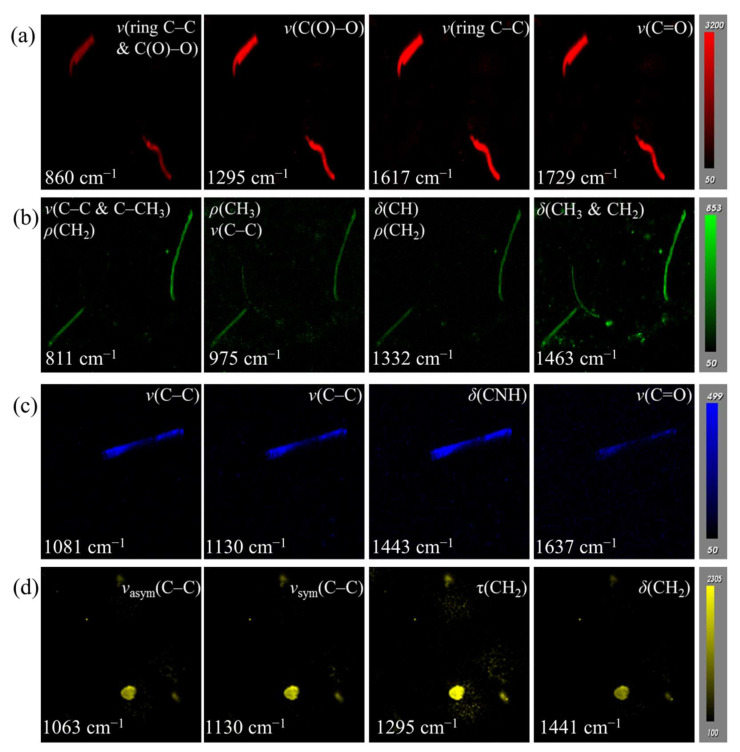
Raman imaging of (**a**) PET, (**b**) PP, (**c**) NY6, and (**d**) PE based on characteristic peaks. *δ*, *v*, *v*_s__ym_, *v*_as__ym_, *ρ*, τ, and *ω* represent the vibration of bending, stretching, symmetric stretching, anti-symmetric stretching, rocking, twisting, and wagging, respectively.

The dominant characteristic peaks in the range of 800 to 1800 cm^−1^ were selected for Raman imaging of MPs. In order to avoid the interference of characteristic peaks of different MPs, four 1200 µm × 1200 µm ROIs were randomly selected for MP mapping of PET, PP, NY6, and PE, respectively. Raman imaging data corresponding to Figure 3a–d were respectively used in the color scale bar to extract Raman signals. The PET mapping of GMF was carried out using peak areas at 860, 1295, 1617, and 1729 cm^−1^, respectively (Figure 3a). The peak area maps in Figure 3a present the consistency of the microplastic position. The image contrast of the peak area imaging at 1617 cm^−1^ was higher than that at 860, 1295, and 1729 cm^−1^ because the strongest Raman peak of PET was located at 1617 cm^−1^ (Figure 2). Therefore, the peak area at 1617 cm^−1^ was selected for PET mapping.

From Figure 3b, the amount and location of PP identified by peak area imaging at 811 cm^−1^ and 1332 cm^−1^ were basically the same, while peak area imaging at 975 cm^−1^ and 1463 cm^−1^ identified more PP, especially the latter. In order to explore the reliability of the peak area imaging to identify PP, the Raman spectra were extracted at the mapped pixels, and then the characteristic peaks were marked (Figure S2). It was found that some of the PP identified at 975 and 1463 cm^−1^ were false positives, mainly due to interference from the background and impurities (Figure S2). In conclusion, the mapping image of 811 cm^−1^ had higher contrast compared with the peak area imaging at 1332 cm^−1^, and fewer disorderly pixels, compared with that at 975 and 1463 cm^−1^. Therefore, it was selected for reliable and rapid identification of PP.

The NY6 and PE mapping at different characteristic peaks were presented in Figure 3c,d, respectively. The morphology features of NY6 and PE identified by peak area imaging at all characteristic peaks were highly consistent. For the mapping of NY6, the imaging results of peak areas at 1081, 1130, and 1443 cm^−1^ were similar and better than at 1637 cm^−1^. However, the peak at 1130 cm^−1^ is a common characteristic peak of NY6 and PE (Figure 2). Thus, it does not have unique characteristics and cannot be used to identify NY6. In addition, the position of the characteristic peak of NY6 at 1443 cm^−1^ was close to that of PE at 1441 cm^−1^, which may cause interference in the calculation of the peak area, resulting in false identification. The characteristic peak 1295 cm^−1^ (twisting of CH_2_) of PE also characterizes the stretching of C(O)–O of PET; thus, it cannot be used to identify MPs. Therefore, 1081 and 1063 cm^−1^ were used as characteristic peaks of NY6 and PE for Raman imaging, respectively.

### 3.3. MP Mapping Using Raman Images Combined with Chemometrics

**Figure 4:**
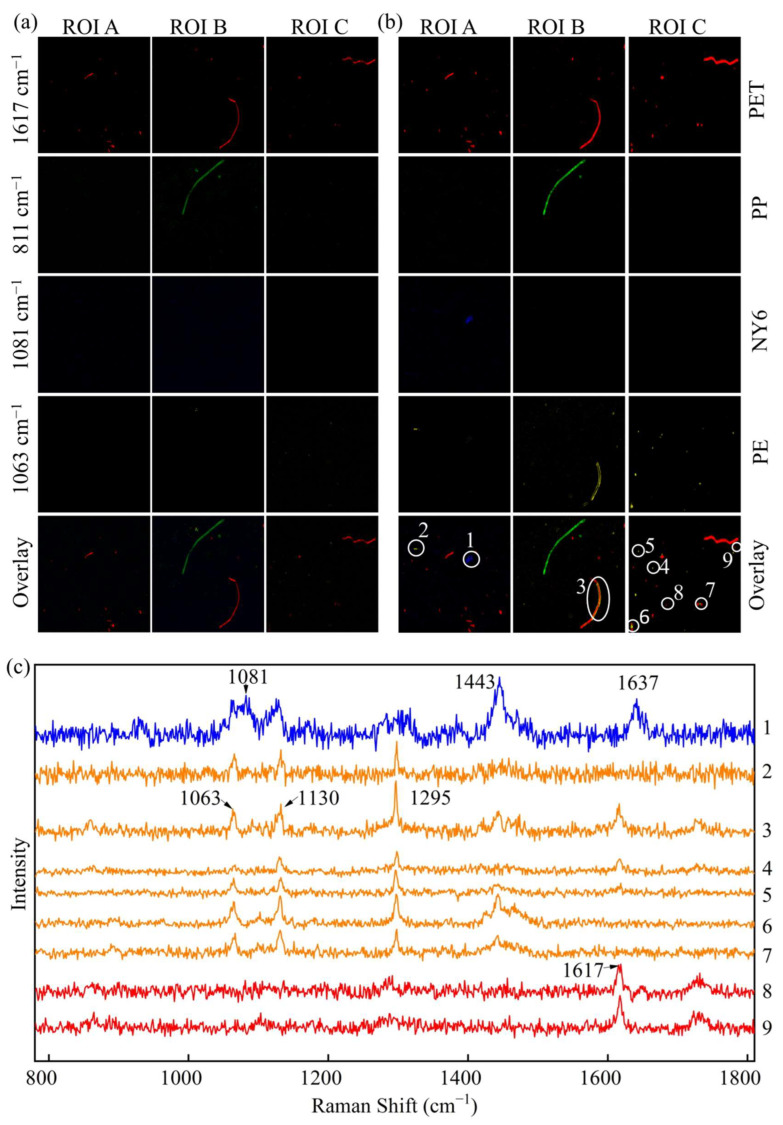
(**a**) Individual Raman maps and overlaid maps obtained by peak area imaging at different characteristic peaks, (**b**) individual Raman maps and overlaid maps obtained by the DCLS method, (**c**) extracted Raman spectra from the circled areas.

According to the given procedure (Figure 1a), a GMF with various MPs was prepared. Three 1200 µm × 1200 µm ROIs were selected randomly from the observation area of the GMF for Raman imaging (Figure 4). Peak area imaging and DCLS methods were proposed to identify MPs in the selected areas, and then the four images were merged as one image for overall MPs identification. In Figure 4, each color represents a different MP (red was PET, green was PP, blue was NY6, and yellow was PE).

#### 3.3.1. MP Mapping by Peak Area Imaging at Single Characteristic Peak

In Raman images obtained by the peak area of characteristic peaks at 1617, 811, 1081, and 1063 cm^−1^, the baseline noise values were less than 50, 50, 50, and 100, respectively (Figure S3a). Therefore, these values were set as the thresholds for segmenting PET, PP, NY6, and PE, respectively. Positions below the threshold were attributable to a black background, and those above the threshold were marked as MPs. The visualization images obtained by threshold segmentation are shown in Figure 4a. As displayed in Figure 4a, three ROIs were simultaneously mapped at different peaks (1617, 811, 1081, and 1063 cm^−1^ for PET, PP, NY6, and PE, respectively). Many MPs of PET were identified in all ROIs, and their morphology features were mainly dominated by tiny particles, and very few were filamentous. The largest MPs had a length of about 620 µm. The MPs of PP were identified in only one of the ROIs, including a filamentous MP with a length of about 840 µm, and two MPs had small particle sizes. Only three fragments of PE were identified, and almost no NY6 was identified using peak area imaging at 1081 cm^−1^. Very few tiny microplastics of NY6 and PE were identified in the first ROI and the second ROI, respectively. It can be seen from the overlay images (Figure 4a) that the MPs of PET and PP have a higher probability of being released from the filter bag, while NY6 and PE have a lower release probability. This may be related to the structure and production of the filter bag.

#### 3.3.2. MP Mapping by Raman Imaging Combined with DCLS

DCLS calculations were performed on three ROIs for the identification of PET, PP, NY6, and PE, respectively. The spectra of the four pure components in Figure 2 were selected and used as reference spectra for DCLS calculations. When the scaling factor of the reference spectrum was greater than 0.1 (the maximum scaling factor is 1), the position was marked as the MPs corresponding to the reference spectrum (Figure S3b). Therefore, the visualization images of PET, PP, NY6, and PE were obtained by segmentation with the threshold of 0.1, respectively, as shown in Figure 4b. The algorithm also generated four images simultaneously and independently after processing. From Figure 4b, the overlaid images clearly display numerous MPs released from filter bags. Comparing the overlay of peak area imaging (Figure 4a) and DCLS calculation (Figure 4b), the distribution and morphology of MPs identified by the two methods were in agreement, but more tiny MPs, such as PET, PE, and NY6, were identified by the DCLS method. In order to verify the reliability of the DCLS method in identifying MPs, the Raman spectra were extracted from the marked pixels, as shown in Figure 4c. The Raman spectrum (marked as 1) had three characteristic peaks at 1081, 1443, and 1637 cm^−1^, which are in perfect agreement with the characteristic peaks of NY6. Similarly, the Raman spectra (marked as 2–7) had three characteristic peaks at 1063, 1130, and 1295 cm^−1^, which are consistent with the characteristic peaks of PE. In addition, there is a prominent Raman peak at 1617 cm^−1^, which is a unique feature of PET. Therefore, the DCLS method more accurately identified MPs, especially tiny ones.

The overlaid maps of the three ROIs were accumulated by ImageJ 1.8 software, and the number of various MPs was calculated separately. The numbers of PET, PP, NY6, and PE MPs identified by the peak area imaging of characteristic peaks were 30, 2, 0, and 3, respectively. The total number of MPs in the three ROIs obtained by the DCLS method was 56, and the numbers of PET, PP, NY6, and PE MPs were 38, 2, 1, and 15, respectively. Compared with peak area imaging, the DCLS method can identify more MPs, especially for the MPs with smaller particle sizes.

For chemical imaging, conventional univariate data analysis, such as peak area imaging, provides a powerful way to reveal the spatial distribution of MPs when enough selective information from single spectral channels is available. However, multivariate data analysis methods based on signals from multiple spectral channels, such as DCLS from chemometrics, are more efficient and reliable for identifying MP features from imaging data when selective information is not provided, or the information acquired by a single spectral channel is insufficient [45]. The Raman imaging with a scanning step of 3 µm and an ROI of 1200 µm × 1200 µm was employed to improve the performance on detection of MPs; the theoretically estimated minimum concentration of detectable MPs was 15. As described above, Raman imaging combined with DCLS calculations can image single- and multi-component MPs.

### 3.4. Simultaneous Identification of Various MPs Released from Filter Bags

According to the procedure described in Figure 1b, 36 GMFs enriched in MPs were obtained. Raman imaging and DCLS calculations were performed on three ROIs selected in GMFs, and then the three images were merged as the final image to simultaneously identify MPs of PET, PP, NY6, and PE. Table 1 illustrates the multiple MPs, including PET, PP, NY6, and PE, present in the soaking solution.

As presented in Table 1, MPs were detected in 34 GMFs, accounting for 94.4% of the total samples, indicating that the filter bags have a very high probability of releasing MPs during the soaking process. For filter bags of the same source, the types of MPs released by the filter bags were nearly the same under almost all soaking conditions. For sample A (non-woven PET), PET was detected in all cases, and PE was also detected in all but five GMFs. This further confirmed that most of the filter bags of sample A were made of PET and PE materials, which is consistent with the previous analysis (Figure 2 and Table S1). The impurity of the PET filter bag may be due to a mixture of PET and PE in the raw material or PE residue in the production line.

For sample B (non-woven PP), PP was identified in all but one of the GMFs, while PET or/and PE were also detected in 2/3 of the total GMFs. This is inconsistent with the previous analysis, which found the filter bag of sample B to be made of only PP material (Figure 2 and Table S1). Interestingly, PP was detected in 83.3% of the GMFs of sample C (woven NY6), whereas NY6 was detected in only one sample. This may be because sample C was woven with NY6 with good willfulness so that it maintained good stability during soaking. In addition, MPs different from the material of the filter bag itself were found in both sample B and sample C. For example, PET and PE were found in sample B, and PP was found in sample C, which may originate from the string (Figure 2).

### 3.5. Exploration of the Origin of MPs from Filter Bags

**Figure 5:**
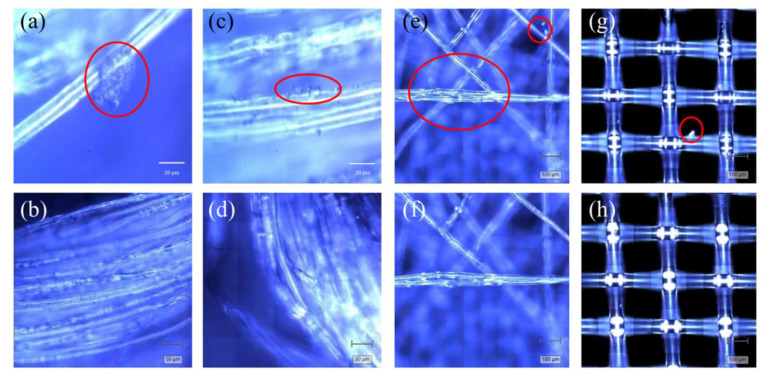
Surface morphologies of the string of samples B (**a**,**b**), the string of sample C (**c**,**d**), the non-woven filter bag (**e**,**f**), and the woven filter bag (**g**,**h**) before and after soaking, respectively.

In order to explain the origin of the MPs, microscopic methods were used to observe the morphological features of the filter bags and strings before and after soaking. The strings of the filter bags were twisted from a large number of fine fibers, and adhered plastic fragments and particles were observed on the surface of the fine fibers, as shown in the circled areas in Figure 5a,c. During high-temperature soaking, the fine fibers and the adhered MPs entered the soaking solution due to the swelling effect of water absorption, such as the MPs (masked 3) shown in Figure 4b,c. In addition, because the MPs on the electrostatic adsorption surface lose their adsorption effect in water, coupled with the scouring effect of water, tiny fragments and particles entered the water phase, and the number of MP particles adsorbed by the fibers was reduced, as shown in Figure 5b,d. This is consistent with our previous results, in which higher numbers of MPs were detected in the filtration residues of the soaking solutions (Figure 4).

In addition, for the two types of filter bags, the micro-morphologies of the non-woven filter bag and the woven filter bag are shown in Figure 5e,g. The non-woven filter bag had a loose structure and was composed of a large number of fine fibers with disordered assembly. During the spinning process, the plastic fibers exhibited crack defects due to stretching (the middle of Figure 5e), and small plastic fragments were also adsorbed on the fibers (the upper right of Figure 5e,f). These fragments detached during the soaking process (Figure 5f,h). Compared with the non-woven filter bag, the structure of the woven filter bag was more stable, with little change during soaking, and the probability of MP release was extremely low, which was consistent with the previous results (Figure 4 and Table 1). However, because the NY6 fragments produced by the spinning process were adsorbed on the surface of the filter bag and released into the soaking solution, only a very small amount of NY6 fragments were identified in the residue of the soaking solution.

The results showed that the filter bag changed to varying degrees during the soaking process. Small substances adhering to the filter bags were easily released into the soaking solution, and the larger fiber plastics also changed due to thermal shock [46]. The MPs adhering to the surface of the tea filter bags were removed by multiple pre-washes, which reduced the amount of MPs entering the tea soup (Figure S4 and Table S2). Therefore, pre-washing is an effective way to reduce the intake of MPs. In comparison, the use of woven NY6 filter bags to subpack tea and coffee has a lower risk of releasing MPs during soaking, and the use of plastic-free strings can also greatly avoid the release of MPs.

## 4. Conclusions

Raman imaging combined with chemometric methods was used to identify MPs released from food filter bags. According to the results, up to 94% of the filter bags released MPs during soaking, and the probability of release was not significantly correlated with temperature and soaking time. The released MPs mainly originated from PET, PP, and PE in non-woven filter bags and strings made of various plastics. The woven NY6 filter bags were the least likely to release MPs. These results suggest that the use of plastic-based food filter bags for sub-packaging of soft drinks can cause potential risks to human health from MP contamination. Overall, the Raman imaging-based MP mapping method is convenient and intuitive for identifying MPs, as well as their distribution. In addition, the preliminary results provide a method to study the release of MPs from filter bags, with important implications for food control and MP regulation.

## Figures and Tables

**Table 1:** Results of simultaneous identification of various MPs released from different filter bags soaked under different conditions.

Soaking Sample Type	Soaking Temperature (°C)	Soaking Time (min)			
		1	5	10	30
A	70	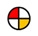	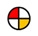	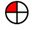	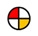
	80	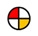	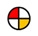	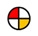	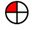
	90	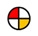	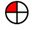	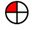	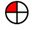
B	70	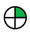	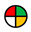	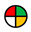	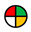
	80	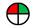	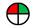	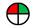	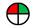
	90	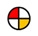	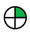	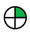	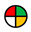
C	70	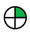	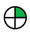	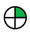	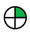
	80	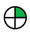	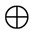	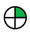	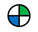
	90	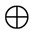	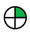	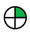	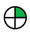

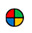
: Red: PET, Green: PP, Blue: NY6, Yellow: PE.

## Supplementary Materials

The following supporting information can be downloaded at: https://www.mdpi.com/article/10.3390/foods11182871/s1, Figure S1: MPs mapping on ROI (420 μm × 210 μm) by Raman imaging with the scanning step of 1, 3, and 5 μm, respectively; Figure S2: The Raman spectrum corresponding to the signal substance in the MPs maps. (a) MPs map obtained by peak area imaging at the peak of 1463 cm^−1^, (b) Spectra of the corresponding to the regions marked in the MPs map; Figure S3: Raman imaging visualization of MPs in the ROI B by peak area imaging at different characteristic peaks (a) and by DCLS method (b); Figure S4: Overlaid maps of MPs in washing solution of tea filter bags obtained by the DCLS method; Table S1: Modes of vibration in the spectrum of sample A, sample B, sample C, PET, PP, NY6, and PE; Table S2: Statistics on the number of MPs in washing solution of different tea filter bags.
