# Supplementary material for: Identification and Evaluation of Microplastics from Tea Filter Bags Based on Raman Imaging
Source: Foods. 2022 Sep 16;11(18):2871. doi: 10.3390/foods11182871 (PMC9497986; doi:10.3390/foods11182871)
Supplement: Supplementary file 1 [file foods-11-02871-s001.zip › foods-1885009-supplementary.pdf]

Supplementary Materials

Identification and evaluation of microplastics from tea filter bags based on Raman imaging

Tingna Mei [1], Jiahua Wang [1,2,*], Xiaofeng Xiao [1], Jingwen Lv [1], Qiaocong Li [1], Huang Dai [1,2], Xiaodan Liu [1,2] and Fuwei Pi [1,3]

[1] College of Food Science and Engineering, Wuhan Polytechnic University, Wuhan 430023, Hubei, People's Republic of China

[2] Hubei Key Laboratory for Processing and Transformation of Agricultural Products (Wuhan Polytechnic University), Wuhan 430023, People's Republic of China

[3] School of Food Science and Technology, Jiangnan University, Wuxi 214122, Jiangsu, People's Republic of China

*Corresponding author. College of Food Science and Engineering, Wuhan Polytechnic University, Wuhan 430023, Hubei, People's Republic of China

E-mail addresses: w.jiahua@163.com (J. Wang)

Table S1. Modes of vibration in the spectrum of sample A, sample B, sample C, PET, PP, NY6 and PE.

Raman shift /cm^{-1}	Group	PET	PP	NY6	PE	Filter bag A	Filter bag B	Filter bag C	String of B	String of C
399	ω(CH$_2$)<br>δ(C–H)		✓				✓			✓
811	v(C–C)<br>v(C–CH$_3$)<br>ρ(CH$_2$)		✓				✓			✓
860	v(ring C–C)<br>v(C(O)–O)	✓				✓			✓	
975	ρ(CH$_3$)<br>v(C–C)		✓				✓			✓
1063	v_{asym}(C–C)				✓	✓			✓	
1081	v(C–C)			✓				✓		
1130	v_{sym}(C–C)			✓	✓	✓		✓	✓	
1295	v(C(O)–O)<br>τ(CH$_2$)	✓			✓	✓			✓	
1332	δ(CH)<br>ρ(CH$_2$)		✓				✓			✓
1441	δ(CH$_2$)				✓					
1443	δ(CNH)			✓				✓		
1463	δ(CH$_3$)<br>δ(CH$_2$)		✓				✓			✓
1617	v(ring C–C)	✓				✓			✓	
1637	v(C=O)			✓				✓		
1729	v(C=O)	✓				✓			✓	
2852	v_{sym}(CH$_2$)				✓	✓			✓	
2885	v_{asym}(CH$_2$)				✓	✓			✓	
2886	v_{sym}(CH$_3$)		✓				✓			✓
2904	v(CH$_2$)			✓				✓		

PET: polyethylene terephthalate, PP: polypropylene, NY6: nylon 6, PE: polyethylene.
δ: bending, v: stretching, v_{sym}: symmetrical stretching, v_{asym}: anti-symmetric stretching, ρ: rocking, τ: twisting, ω: wagging.

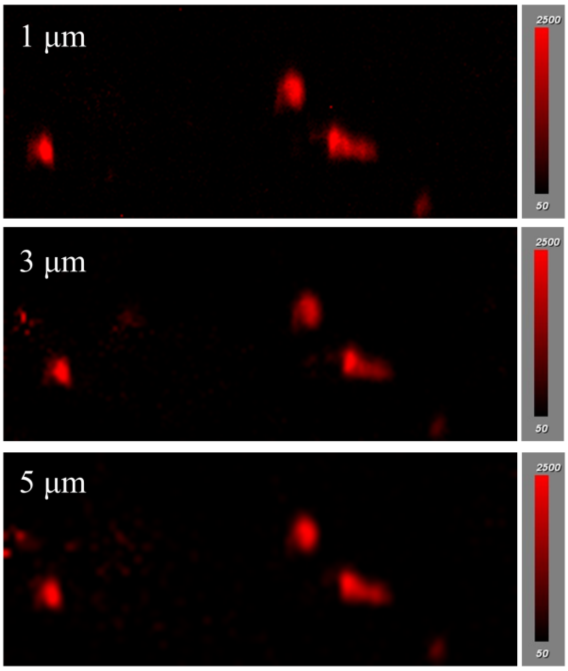

Figure S1. MPs mapping on ROI (420 μm × 210 μm) by Raman imaging with the scanning step of 1, 3, and 5 μm, respectively.

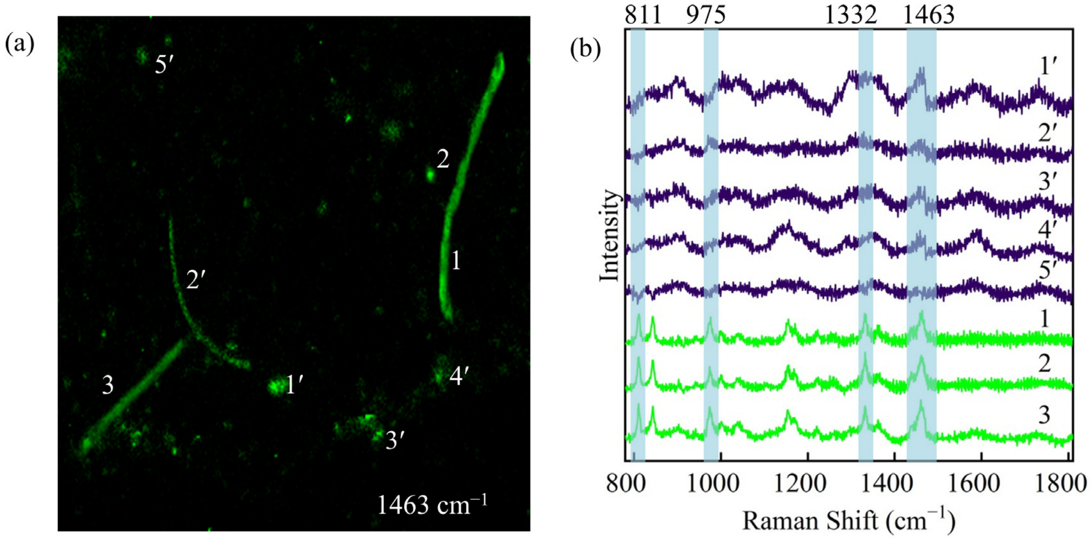

Figure S2. The Raman spectrum corresponding to the signal substance in the MPs maps. (a) MPs map obtained by peak area imaging at peak of 1463 cm^{-1}, (b) Spectra of the corresponding to the regions marked in in the MPs map.

Raman maps were susceptible to interference from background signals. For example, the spectrum shown in Figure S2 has strong interference in the cluttered pixel at 1463 cm^{-1}. The Raman spectra of pixels labeled 1'–5' did not show the characteristic peaks of plastic, which is a false positive result. In the other pixels (labeled 1–3), the Raman peaks were located at 811, 975, 1332, and 1463 cm^{-1}, which matched with peaks of PP. This was also the reason why 1463 cm^{-1} was not selected as the characteristic peak for Raman imaging.

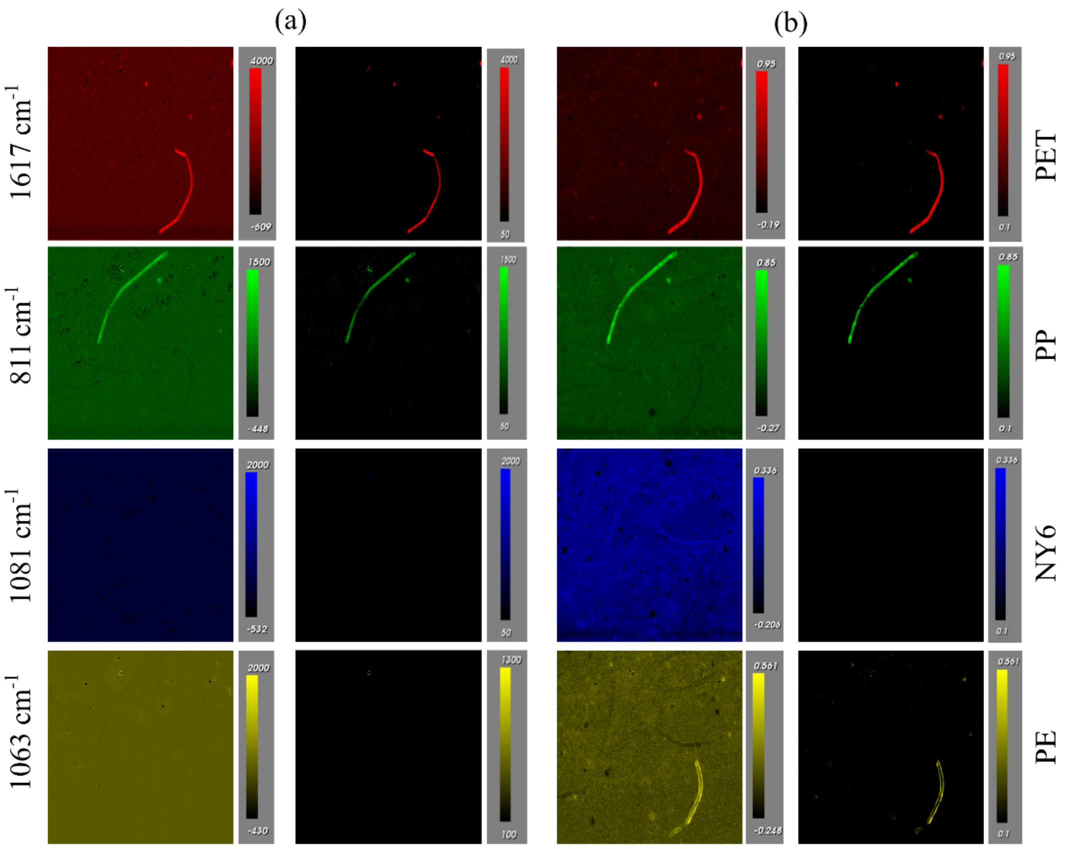

Figure S3. Raman imaging visualization of MPs in the ROI B by peak area imaging at different characteristic peaks (a) and by DCLS method (b).

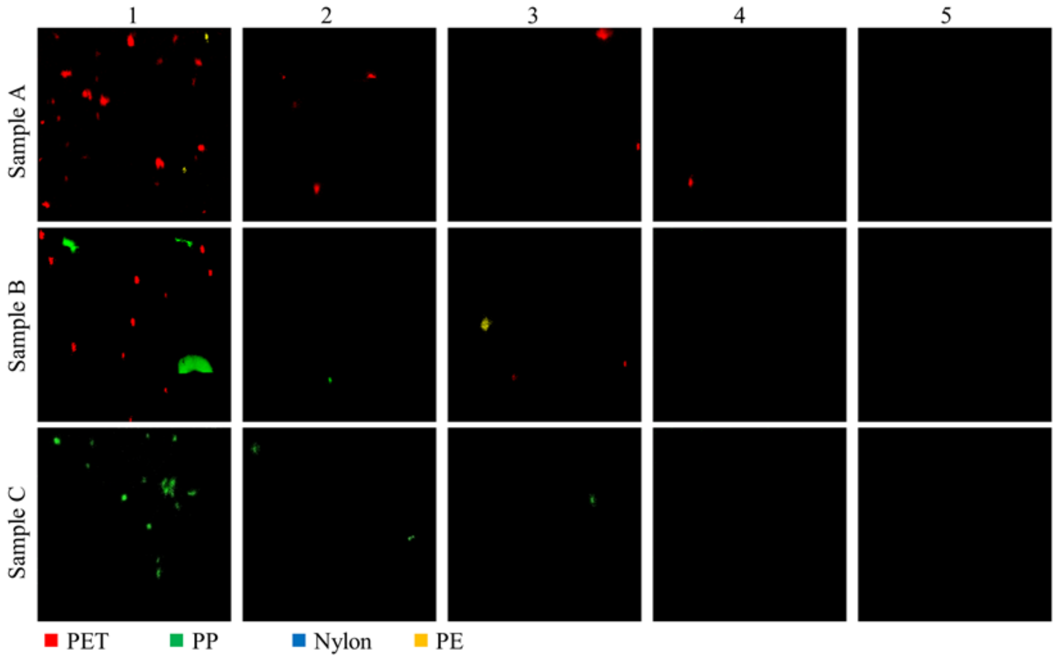

Figure S4. Overlaid maps of MPs in washing solution of tea filter bags obtained by the DCLS method.

Table S2. Statistics on the number of MPs in washing solution of different tea filter bags.

Washing times	1	2	3	4	5
sample A	27	4	2	1	0
sample B	14	1	3	0	0
sample C	12	2	1	0	0

The filter bags were put into a 30 mL glass cone bottle with ultrapure water and were stirred with the stirring speed of 5 r/s for 10 s. And then, the filter bags were taken out and were put it into the next same glass cone bottle for washing. Repeat the above process 5 times. The washing solution was filtered in turn by GMF. The Raman imaging results shown that the number of detected MPs decreased with increasing washing times (Figure S4). As presented in Table S2, the MPs adhered on the surface of the tea filter bags were almost completely eluted after one pre-wash. Although no MP was observed after 5 washes, this does not mean that the tea filter bags no longer

released smaller MPs during soaking. Therefore, pre-washing is an effective way to reduce the intake of MPs.
